# A New Approach for the Determination of Ammonite and Nautilid Habitats

**DOI:** 10.1371/journal.pone.0087479

**Published:** 2014-01-27

**Authors:** Isabelle Kruta, Neil H. Landman, J. Kirk Cochran

**Affiliations:** 1 Division of Paleontology American Museum of Natural History, New York, New York, United States of America; 2 Department of Geology and Geophysics, Yale University, New Haven, Connecticut, United States of America; 3 School of Marine and Atmospheric Sciences, Stony Brook University, Stony Brook, New York, United States of America; University of Maryland, United States of America

## Abstract

Externally shelled cephalopods were important elements in open marine habitats throughout Earth history. Paleotemperatures calculated on the basis of the oxygen isotope composition of their shells can provide insights into ancient marine systems as well as the ecology of this important group of organisms. In some sedimentary deposits, however, the aragonitic shell of the ammonite or nautilid is poorly or not preserved at all, while the calcitic structures belonging to the jaws are present. This study tests for the first time if the calcitic jaw structures in fossil cephalopods can be used as a proxy for paleotemperature. We first analyzed the calcitic structures on the jaws of Recent *Nautilus* and compared the calculated temperatures of precipitation with those from the aragonitic shell in the same individuals. Our results indicate that the jaws of Recent *Nautilus* are secreted in isotopic equilibrium, and the calculated temperatures approximately match those of the shell. We then extended our study to ammonites from the Upper Cretaceous (Campanian) Pierre Shale of the U.S. Western Interior and the age-equivalent Mooreville Chalk of the Gulf Coastal Plain. In the Pierre Shale, jaws occur *in situ* inside the body chambers of well-preserved *Baculites* while in the Mooreville Chalk, the jaw elements appear as isolated occurrences in the sediment and the aragonitic shell material is not preserved. For the Pierre Shale specimens, the calculated temperatures of well-preserved jaw material match those of well-preserved shell material in the same individual. Analyses of the jaw elements in the Mooreville Chalk permit a comparison of the paleotemperatures between the two sites, and show that the Western Interior is warmer than the Gulf Coast at that time. In summary, our data indicate that the calcitic jaw elements of cephalopods can provide a reliable geochemical archive of the habitat of fossil forms.

## Introduction

Ammonoids, an extinct class of cephalopods, constitute one of the best documented fossil groups [1]. They are restricted to a marine habitat and exhibit a broad geographic and stratigraphic range. Because the shell is composed of calcium carbonate, ammonites can be used to provide insights into ancient seawater temperatures [2], [3], [4], [5], [6], [7], [8], [9]. Calculation of paleotemperatures also provides information about the ecology (depth distribution and habitat) of this important group of organisms [9], [10], [11]. Studies of the stable isotope composition of the Recent ectocochleate cephalopod *Nautilus* have demonstrated that the δ^18^O of the aragonitic shell accurately reflects the temperature of the sea water during the secretion of the shell [12], [13]. [14], [15], [16], [17]. This relationship is assumed to apply as well to externally shelled fossil cephalopods such as ammonites and nautilids [9], [18], [19], [20]. A prerequisite for the use of fossil shell material in such an analysis is that the shell must be well preserved [21]. In some sedimentary deposits, however, the aragonitic shell of the ammonoid or nautilid is poorly or not preserved at all, while the calcitic structures belonging to the jaws are present (e.g., Jurassic Solnhofen Plattenkalk from Germany; Lower Cretaceous Bassin Vocontien from France). Previous studies have used ammonite jaw material as a proxy for paleotemperature [22], [23], but it is unclear in these studies if 1) these structures were secreted in isotopic equilibrium with sea water (defined as the same temperature-dependent fractionation of aragonite/calcite relative to water as that of other aragonite/calcite secreting mollusks) and 2) the state of preservation was sufficiently adequate to retain the original isotopic composition. The aim of this study is to test the hypothesis that unaltered calcitic jaw structures from fossil ammonites can be used to reconstruct paleotemperatures. To begin, we analyzed the calcitic structures on the upper and lower jaws of Recent *Nautilus* and compared the calculated water temperatures with those of the aragonitic shell in the same individuals. The shell and jaws do not exhibit the same mineralogy (aragonite versus calcite), and are not secreted by the same tissue, thus representing independent systems. We then extended our studies to fossil *Baculites* from the Upper Cretaceous of North America. Our studies reveal that both the outer shell and the calcitic jaw elements in fossil ammonites yield reliable sea water temperatures provided that both features are well preserved and retain the original mineralogy and microstructure.

## Materials and Methods

### Ethics statement

The species of *Nautilus pompilius* (Mollusca: Cephalopoda) is not endangered or protected. The specimens of *Nautilus* were collected with the approval of the Department of Fisheries and Environment Unit of Vanuatu and imported to the American Museum of Natural History with the authorization of the U.S. Fish and Wildlife Service. Copies of the permits are held by the American Museum of Natural History (AMNH) where the specimens are deposited.

### Nautilus

**Figure 1:**
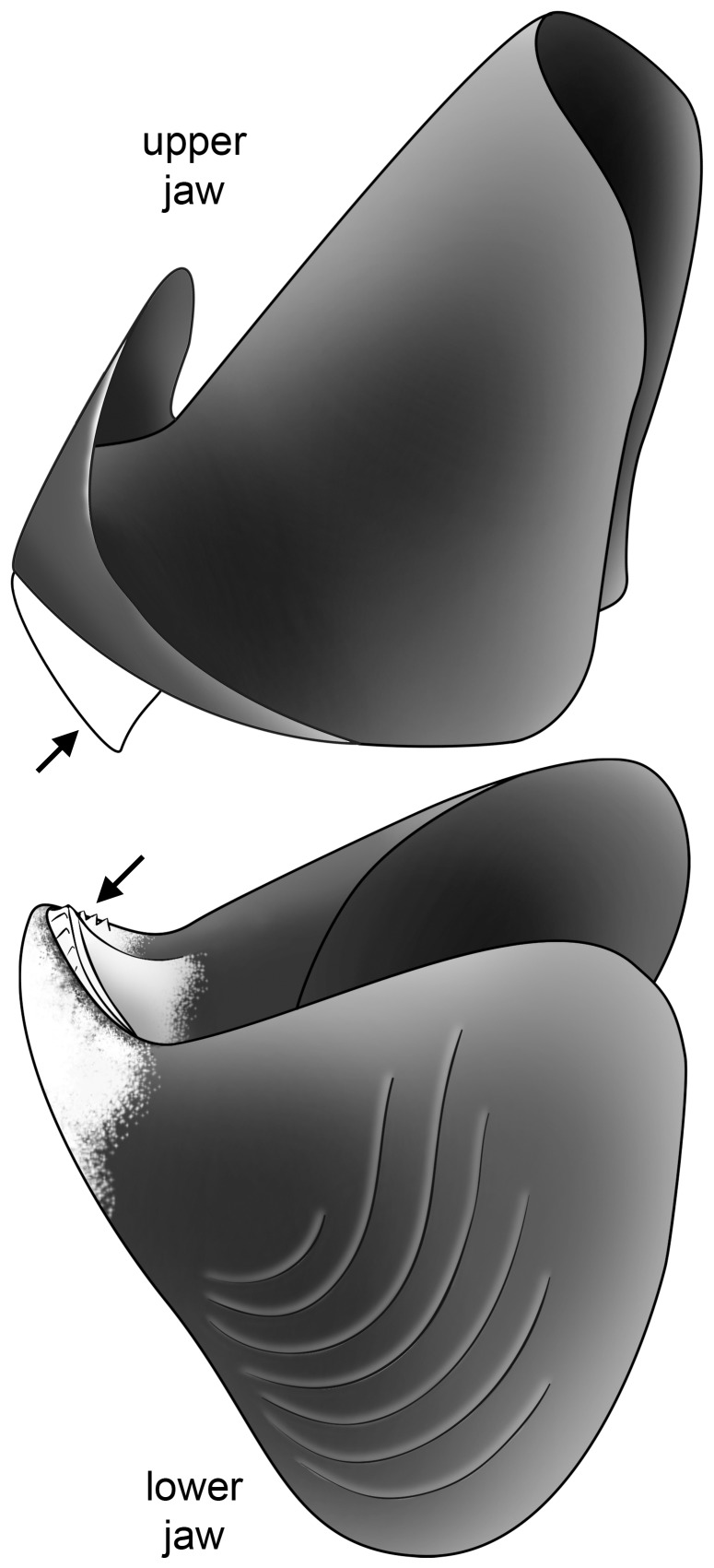
Lateral view of the upper and lower jaws of *Nautilus pompilius*. Calcitic structures are represented in white, while the main chitinous part of the jaws is displayed in black. Arrows indicate where samples were taken for isotopic analyses.

We sampled eight specimens of *Nautilus pompilius* from Vanuatu captured in July 2004. All specimens are mature individuals and both sexes are represented. The aragonitic shell and the calcitic coverings of the chitinous jaws were sampled for each individual. The outer shell wall was sampled at the aperture of the body chamber. The rostrum of the upper jaw bears a thick arrowed-shaped calcitic structure called the rhyncholite. The calcitic covering of the lower jaw, which is thinner than the rhyncholite, features calcitic denticules on the oral surface and is called the conchorhynch (Fig. 1). Both calcitic structures were sampled and the isotopic results are listed in Table 1. The rhyncholite and conchorhynch are mostly composed of calcite, but a thin aragonitic layer appears at the contact beween the conchorhynch/rhyncholite and the chitinous part of the jaw [24]. In order to avoid contamination with this aragonitic layer, we sampled the anterior tip of the rhyncholite on the upper jaw and the denticles and the inner part of the conchorhynch on the lower jaw (Fig. 1). The samples were powdered and treated following the method in Allmon *et al.* [25] in order to remove the organic material present in the shell and in the lower jaw. The samples were washed in 15% H_2_O_2_ for three hours. The H_2_O_2_ was then pipeted off and flushed three times with methanol (99.9%).

**Table 1:** Isotope data for *Nautilus*.

Recent *Nautilus* (Vanuatu)	Sample	Specimen number	Mineralogy	δ^18^O† (‰)	δ^13^C† (‰)	T (°C )
*Nautilus pompilius*	shell	AMNH 310420	aragonite	0.95	2.12	18.3
*"*	rhyncholite	"	calcite	−0.10	0.06	18.2
*"*	conchorhynch	"	calcite	−0.02	−0.49	17.8
*"*	shell	AMNH 310433	aragonite	0.77	1.50	19.1
*"*	conchorhynch	"	calcite	−0.34	0.36	19.3
*"*	shell	AMNH 310434	aragonite	0.99	2.22	18.1
*"*	conchorhynch	"	calcite	−0.23	0.06	18.8
*"*	shell	AMNH 310435	aragonite	0.89	1.54	18.6
*"*	rhyncholite	"	calcite	−0.46	0.05	19.8
*"*	shell	AMNH 310432	aragonite	1.28	1.71	16.7
*"*	rhyncholite	"	calcite	−0.37	0.3	19.4
*"*	shell	AMNH 310421	aragonite	1.33	1.47	16.5
*"*	rhyncholite	"	calcite	−0.07	0.64	18.1
*"*	shell	AMNH 310422	aragonite	1.47	1.63	15.8
*"*	rhyncholite	"	calcite	−0.51	0.41	20.0
*"*	shell	AMNH 310431	aragonite	1.77	1.36	14.4
*"*	rhyncholite	"	calcite	−0.27	−0.8	19
*Mean* ± *1*σ *shell T*						*17.2*±*1.6*
*Mean* ± *1*σ *jaw T*						*18.9*±*0.8*

†Values relative to VPDB.

### Fossil material

**Figure 2:**
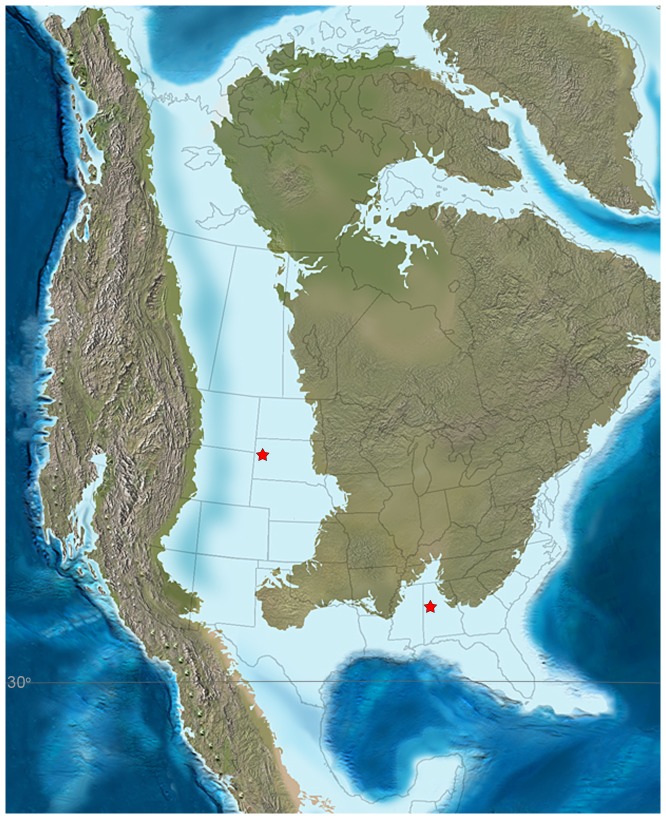
Paleomap of North America during the Late Cretaceous (85Ma, from Blakey, 2011). Fossil localities in South Dakota and Alabama are indicated by red stars. The locality in South Dakota was within the Western Interior Seaway, while the locality in Alabama was on the Gulf Coast. The paleolatitude has been estimated from Smith *et al.* [34].

**Figure 3:**
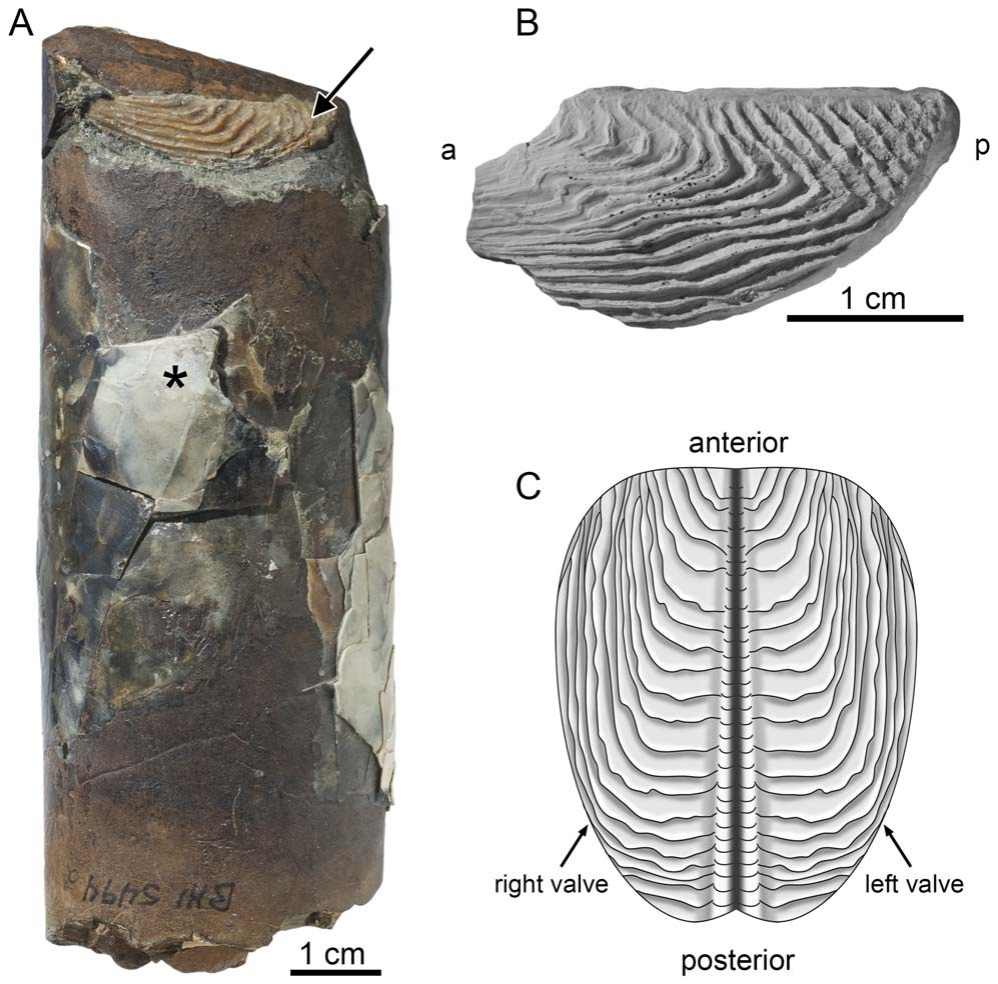
Ammonite shell material and aptychus jaw element. A) *Baculites* sp. (smooth) from the Pierre Shale, Butte County, South Dakota, with aptychus (arrow) preserved in the body chamber of the ammonite. Pieces of the aragonite shell (asterisk) are present on the steinkern. B) Reconstruction of the aptychus type of jaw in *Baculites*. The lower jaw is covered by two calcitic valves that usually separate after death and are found isolated in the sediment. C) Aptychus from the Mooreville Chalk, Alabama. Isolated valves are present in the sediment and are attributed to *Baculites* sp. (smooth). a-anterior, p-posterior.

The first set of ammonite samples consists of the outer shell wall as well as jaw elements of *Baculites* sp. (smooth) from the Upper Cretaceous (lower Campanian) Gammon Ferruginous Member of the Pierre Shale, Butte County, South Dakota (Fig. 2) (see [26] for locality information). This locality was selected based on the abundance of extremely well preserved specimens of *Baculites* (Fig. 3A) with pieces of the aragonitic nacreous shell material still attached to the steinkern (composite internal molds) and jaw elements preserved inside the body chamber of the same individuals (*in situ*). While the shell is quite commonly preserved at this locality, jaws inside the body chamber are rare [26] and only seven specimens with both shell and jaw could be sampled for our analysis. The jaw material consists of the calcitic covering of the lower jaw, called the aptychus (Fig. 3B). For comparison, we also analyzed well-preserved shell material from two specimens of *Baculites* sp. (AMNH 78053 and 51754) without jaws inside the body chamber.

The second set of samples consists of aptychi (i.e. the calcitic valves of the lower jaws) preserved loose in the sediment from the time-equivalent lower Campanian Mooreville Chalk, Greene County, Alabama (see [26] for a discussion of the stratigraphic relationships). No outer shell wall material is present at this locality and only the calcitic aptychi are preserved (X-ray diffraction analysis in [26]). The aptychi are attributed to *Baculites* sp. (smooth) by comparison with the jaws preserved inside the body chambers of this species in South Dakota [26]. The calcitic aptychi were sampled for isotopic composition.

**Figure 4:**
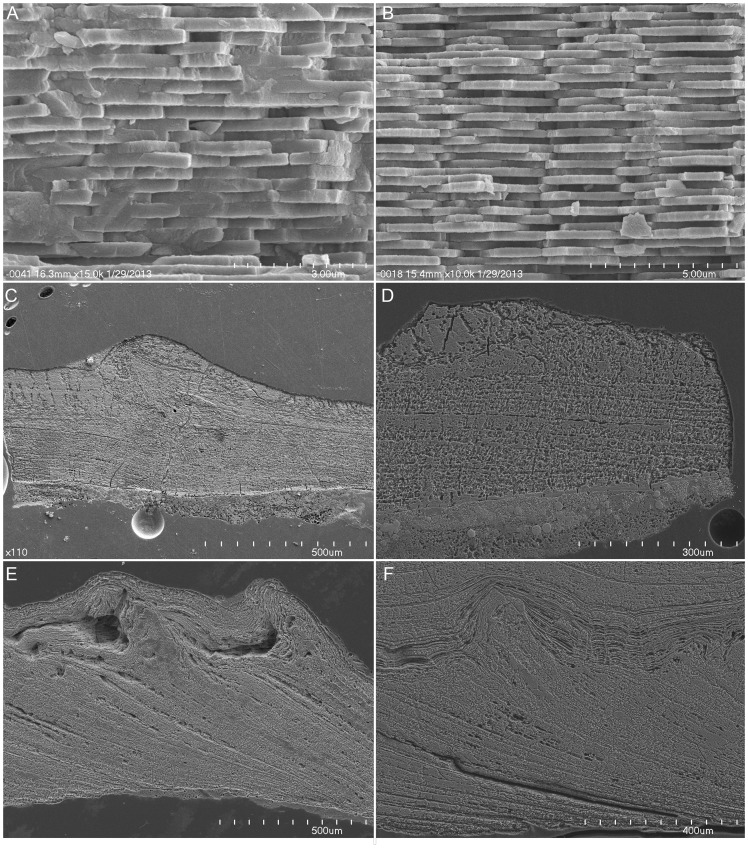
SEM micrographs of the shell and aptychi used to evaluate the Preservation Index. A–B) Nacreous shell in BHI 5146 (A) and AMNH 78053 (B). C–D) Preservation of the aptychi *in situ* in BHI 5146, and close up (D) showing the calcitic increments. E–F) Preservation in the aptychi from the Mooreville Chalk, showing the differentiated layers (R1 and R2) and the calcitic increments in AMNH 66354 (F) and AMNH 66357 (F).

Subsamples of the shell were examined under the SEM (Fig. 4) in order to evaluate the microstructure and assign each sample a Preservation Index (PI) ranging from 1 (poor) to 5 (excellent) for the preservation of the nacreous shell wall [21]. We developed a new approach to evaluate the state of preservation of the aptychi. The Preservation Index for the aptychi is based on the quality of preservation of the microstructure. Because Recent material is unavailable for comparison, we selected an example of the best preserved aptychus (AMNH 54277) from the lower Campanian of Alabama. In AMNH 54277, the calcitic increments are identifiable and the main lamellar layer (R1) and the outer layer (R2) are well defined in this specimen (see [27] for a discussion of aptychus microstructure). We assigned a high Preservation Index (5 =  excellent preservation) if the two layers (R1 and R2) could be identified and/or if calcitic increments could be observed (for very small pieces of aptychus, the outer layer was not always present). A low Preservation Index indicates specimens with massive calcite without any indication of layers. Aptychi samples for SEM analyses were embedded in epoxy, ground, polished, and etched with EDTA from 2 to 5 minutes. Preservation Index values of the outer shell and aptychus are listed in Table 2. To prepare the specimens for analysis, the surfaces of the aptychi from the Mooreville Chalk were scraped to remove extraneous material and then cleaned with a sonifier, and sampled under the microscope. The fossil material is reposited at the American Museum of Natural History (AMNH). Additional material is reposited at the Black Hills Institute of Geological Research (BHI), Hill City, South Dakota.

**Table 2:** Isotope data for ammonites.

Locality Mooreville Chalk	Sample	Specimen	Mineralogy	δ^18^O† (‰)	δ^13^C† (‰)	T (°C)	PI
*Baculites* sp.	aptychus	AMNH 66353	calcite	−2.67	−1.99	24.2	3.5
"	aptychus	AMNH 66354	calcite	−2.07	−2.41	21.6	4
"	aptychus	AMNH 66355	calcite	−2.13	−1.42	21.8	4
"	aptychus	AMNH 66356	calcite	−2.27	−1.7	22.5	4
"	aptychus	AMNH 66357	calcite	−2.16	−1.4	22.0	4.5
*Mean* ± *1σ jaw T*						*22.4*±*1.1*	
Locality Pierre Shale							
*Baculites* sp.	shell	AMNH 51329	aragonite	−2.28	−2.15	27.8	4
*"*	aptychus *in situ*	"	calcite	−3.52	−8.94	27.9	3
*"*	shell	AMNH 66255	aragonite	−1.71	−0.36	25.1	3.5
*"*	aptychus *in situ*	"	calcite	−4.62	−6.62	32.7	2
*"*	shell	AMNH 64489	aragonite	−1.63	−0.04	24.8	3
*"*	aptychus *in situ*	"	calcite	−3.60	−4.39	28.3	1
*"*	shell	BHI 5143	aragonite	−1.66	−3.97	24.9	2.5
*"*	aptychus *in situ*	"	calcite	−3.57	−2.54	28.1	2.5
*"*	shell	BHI 5146	aragonite	−2.46	−1.73	28.6	4
*"*	aptychus *in situ*	"	calcite	−3.54	−3.99	28.0	4
*"*	shell	BHI 5491	aragonite	−3.29	0.69	32.5	2
*"*	aptychus *in situ*	"	calcite	−3.84	−0.85	29.3	2.5
*"*	shell	BHI 5494	aragonite	−1.45	−0.60	23.9	3
*"*	aptychus *in situ*	"	calcite	−4.22	−4.97	31.0	1
*"*	shell	AMNH 78053	aragonite	−2.45	−1.03	28.6	4.5
*"*	shell	AMNH 51754	aragonite	−2.23	−0.06	27.6	4.5
*Mean* ± *1σ shell T (PI>3)*						*27.5*±*1.4*	
*Mean jaw T (PI≥3)*						*28.0*	

†Values relative to VPDB.

### Isotopic analyses

The isotopic analyses were performed at the Keck Paleoenvironmental & Environmental Stable Isotope Laboratory at the University of Kansas (KPESIL). All samples were reacted with phosphoric acid to release CO_2_, which was then analyzed for C and O isotopes using a Thermo Finnigan dual inlet MAT253 isotope ratio mass spectrometer (IRMS). Three standards were used- NIST (National Institute of Standards) NBS-18, NBS-19, and an internally calibrated calcite standard-which were included with each run in order to generate a three point calibration curve to the VPBD scale. A fourth standard, NIST 88b (dolomitic limestone), was used for quality control.

The isotopic temperatures were calculated using the equation of Grossman and Ku [28] for aragonite:
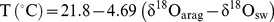
(1)and the equation of O’Neil *et al*. [29] for calcite: 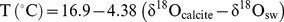
(2)where T is the temperature of the water in which the carbonate precipitated (°C), δ^18^O_arag_ and δ^18^O_calcite_ are the values of δ^18^O for the calcium carbonate (VPDB), and δ^18^O_sw_ is the value of δ^18^O of the seawater (SMOW), respectively. For the *Nautilus* material we used a value of δ^18^O_sw_ of +0.2‰ for seawater at Vanuatu [http://data.giss.nasa.gov/o18data/], and for the fossil material we used a value of δ^18^O_sw_ of -1‰ for Cretaceous seawater [30], [31].

## Results

### Data from Recent Nautilus

The results for *Nautilus pompilius* are reported in Table 1. The values of δ^18^O were converted to temperature following equations (1) and (2). The temperatures calculated for a rhyncholite and a conchorhynch from the same specimen are nearly the same, the difference being only 0.4°C. Thus, the two elements are considered as recording the same temperature. The calculated temperatures of the calcitic jaw elements range from 17.8°C to 20°C, averaging 18.9°C±0.8°C (1σ). Compared with the temperatures of the calcitic jaw elements, those of the outer shell are more variable. The temperatures of the outer shell range from 14.4°C to 19.1°C, averaging 17.2°C±1.6°C.

The values of δ^13^C are listed in Table 1. The carbon isotope composition of the outer shell ranges from 1.4‰ to 2.2‰. The values of δ^13^C of the jaw elements are lighter and range from −0.8‰ to 0.6‰. The conchorhynch and rhyncholite sampled in the same specimen show slightly different values of δ^13^C (−0.5‰ and 0.1‰, respectively).

### Data from the fossil record

The Preservation Index was assigned to the aragonitic shell of *Baculites* sp. (smooth) following Cochran *et al*. [21] and to aptychi following the criteria outlined in the methods section (Fig. 4). The results are summarized in Table 2. In the specimens of *Baculites* sp. (smooth) with both the outer shell and aptychi from South Dakota, the outer shell shows a wide range of PI from 2 to 4.5 while the aptychi are generally not well preserved (only one specimen with PI = 4). In contrast, the aptychi from the Moorville Chalk are very well preserved (PI = 3.5–4.5).

The calculated temperatures are listed in Table 2. The temperatures of the outer shell in *Baculites* sp. (smooth) from the Pierre Shale range from 23.9°C to 32.5°C. Cochran *et al.* [21] documented changes in δ^18^O and δ^13^C as preservation declined, with more poorly preserved shells showing lighter values. Samples with Preservation Index >3 (good) recorded the original isotopic composition and thus, paleotemperature [21]. In specimens of *Baculites sp.* (smooth) with PI >3, the mean temperature of the outer shell is 27.5±1.4°C. The calculated temperatures of the aptychi range from 27.9°C to 32.7°C. In the best preserved aptychus (BHI 5146), the calculated temperature is 28°C. The closest match (difference ≤0.6°C) between outer shell and jaw temperatures appears in the two specimens (BHI 5146 and AMNH 51329) with the best preserved outer shell and aptychi (PI for shell = 4.5, PI for aptychi = 3–4). In BHI 5146 the temperatures of the shell and aptychus are 28.6°C and 28°C, respectively. In AMNH 51329 the temperatures of the shell and aptychus are 27.8°C and 27.9°C, respectively. The aptychi of specimens from the Mooreville Chalk are all well preserved (PI>3.5), and the calculated temperatures range from 21.6°C to 24.2°C, with a mean of 22.4±1.1°C.

The values of δ^13^C of the outer shell in *Baculites* sp. (smooth) from the Pierre Shale range from −4.0‰ to 0.7‰, with a mean of −1.0‰. The highest value (0.7‰) is from a specimen (BHI 5491) with the poorest preservation (PI = 2). The δ^13^C values of the aptychi are generally lighter than those of the outer shell and range from −8.9‰ to −0.8‰. The values of δ^13^C of the aptychi from the Mooreville Chalk are heavier than those from the Pierre Shale and range from −2.4‰ to −1.4‰.

## Discussion

### Isotopic values of Nautilus pompilius

In most of the specimens, the calculated temperatures of the outer shell and calcitic jaw elements match. The average temperature of the outer shell is 17.2±1.6°C and that of the jaw is 18.9±0.8°C. Within the uncertainties, these temperatures are in good agreement. Previous studies have demonstrated that the outer shell is secreted in equilibrium with seawater [15], [16]. Despite the difference in mineralogy, our results suggest that the jaw is also secreted in isotopic equilibrium, and that the temperature calculated for the upper and the lower jaw is the same.

The slight differences in the temperatures calculated between the jaw and outer shell may reflect differences in time averaging. The shell samples are from the aperture and thus represent a finite period of time. Although no data are available on the growth rate of the calcitic jaw elements, the rhyncholite and conchorhynch undoubtedly represent a longer period of time integrating over nearly the entire lifetime of the animal.

Using the temperature-depth profile in Vanuatu (obtained from NOCD database, cruises ID PA-127, PA-165), the depths that correspond to the calculated temperatures of the specimens can be determined. Our results suggest that the outer shell and jaw temperatures correspond to depths of 254–360 m. These values are consistent with habitat depth records of *Nautilus pompilius* elsewhere [32].

The carbon isotopic composition of the outer shell and the jaw elements is more difficult to interpret as several parameters could be involved. The two structures are secreted by independent tissue system. The outer shell is secreted by the mantle whereas the jaws are secreted by tissue in the buccal mass. Therefore, different sources of carbon (DIC and possibly diet) could be incorporated during secretion.

### Isotopic values of *Baculites* sp. (smooth)

The isotopic values in the fossil material reflect the quality of preservation of the samples. In *Baculites* sp. (smooth) from the Pierre Shale of South Dakota, samples of well-preserved outer shell (PI>3; table 2 ) yield a mean temperature of 27.5±1.4°C. The sample with the best preserved outer shell and aptychus is BHI 5146. In this specimen, the calculated temperature of the aptychus (28.6°C) is comparable to that of the outer shell (28°C). The calculated temperature of this aptychus also agrees well with the mean temperature for the other shell samples with PI>3, i.e. 27.5±1.4°C. These observations imply that if the aptychus is well preserved, its δ^18^O-derived temperature matches that of the outer shell. The fossil aptychi from the Mooreville Chalk are generally well preserved (3.5≤PI≤4.5). The two layers (R1 and R2) can be identified as well as the calcitic increments. The calculated temperatures are very consistent, with a mean of 22.4±1.1°C.

### Application to Late Cretaceous paleoenvironments

Our study of the temperatures derived from the outer shell and jaw elements of *Baculites* sp. (smooth) in the Western Interior and U.S. Gulf Coastal Plain provides an example of the advantage of using either the carbonate from the shell and the jaw to determine paleotemperatures. In particular during the early Campanian, the Western Interior has abundant well-preserved outer shell material of ammonites, while the Gulf Coastal Plain has well-preserved aptychi but no comparable shell material. Previous studies have suggested that the two localities are time equivalent (see [26] for discussion on the stratigraphic relationships of the two sites) and our results reveal different temperatures for the two sites. In the Western Interior, the temperatures calculated from well-preserved shell and aptychi average 27.5±1.4°C (n = 6) and 28°C (n = 2), respectively. The temperatures calculated from the aptychi on the Gulf Coastal Plain are lower (22.4±1.1°C; n = 5) despite being at a lower paleolatitude (Fig. 2). Similarly elevated temperatures for the Western Interior have previously been reported for the late Campanian of South Dakota [33]. These differences probably reflect variation in paleogeography between the restricted Western Interior Seaway and the open Gulf Coast. Indeed, Dennis *et al.* [31] used clumped isotopes to document cooler temperatures for the open ocean along the Atlantic Coastal margin (Severn Formation, Maryland) relative to the Western Interior Seaway during the late Maastrichtian. However, some of the differences in temperature we observe between the Western Interior and Gulf Coast during the early Campanian may be due to differences in the isotopic composition of the water at the two environments (δ^18^O_w_). Additional work using clumped isotopes might further tease apart the factors responsible for the differences in temperature between the two sites.

## Conclusion and Perspectives

Our data demonstrate that the temperatures recorded in the shells of Recent *Nautilus pompilius* match the temperatures of the jaw elements in the same individuals. Calcitic structures of the jaws may thus provide a reliable geochemical archive of the habitat of nautilids and, by extension, ammonites. In ammonites, if the aptychus is well preserved, it records the same temperature as well-preserved outer shells. Data from jaw elements are especially valuable for localities where the aragonitic shell of the ammonites is not preserved. Discrepancies in the calculated temperatures of the outer shell and jaw elements in *N. pompilius* are explainable as reflecting differences in time averaging, with the jaw integrating over a longer portion of the lifetime of the animal while individual samples from the outer shell or aperture are restricted in time. The carbon isotopic composition of the jaws is lighter than that of the shell in *N. pompilius* and may reflect differences related to the source of carbon. If so, such studies on ammonites may yield clues into the diet of these extinct animals.
